# Partial volume correction for PET quantification and its impact on brain network in Alzheimer’s disease

**DOI:** 10.1038/s41598-017-13339-7

**Published:** 2017-10-12

**Authors:** Jiarui Yang, Chenhui Hu, Ning Guo, Joyita Dutta, Lucia M. Vaina, Keith A. Johnson, Jorge Sepulcre, Georges El Fakhri, Quanzheng Li

**Affiliations:** 1 Boston University, Department of Biomedical Engineering, Boston, 02215 USA; 2 Massachusetts General Hospital, Department of Radiology, Boston, 02114 USA; 3 Harvard Medical School, Department of Radiology, Boston, 02115 USA; 4 University of Massachusetts Lowell, Department of Electrical and Computer Engineering, Lowell, 01854 USA

## Abstract

Amyloid positron emission tomography (PET) imaging is a valuable tool for research and diagnosis in Alzheimer’s disease (AD). Partial volume effects caused by the limited spatial resolution of PET scanners degrades the quantitative accuracy of PET image. In this study, we have applied a method to evaluate the impact of a joint-entropy based partial volume correction (PVC) technique on brain networks learned from a clinical dataset of AV-45 PET image and compare network properties of both uncorrected and corrected image-based brain networks. We also analyzed the region-wise SUVRs of both uncorrected and corrected images. We further performed classification tests on different groups using the same set of algorithms with same parameter settings. PVC has sometimes been avoided due to increased noise sensitivity in image registration and segmentation, however, our results indicate that appropriate PVC may enhance the brain network structure analysis for AD progression and improve classification performance.

## Introduction

Alzhermer’s disease (AD) is the most common form of dementia. The estimated population affected by AD is more than 30 million people worldwide, which is expected to quadruple over the next 40 years^1^. Extensive evidence has indicated that the pathological hallmarks of AD may be amyloid plaques and neurofibrillary tangles, however, the underlying disease mechanism remains unknown. Moreover, there are currently no treatments of AD that have been proven effective^2–5^. The hippocampus is an intricate region located in the medial temporal lobe of brain, which has been suggested playing an important role in cognitive learning and memory processes. It is well established that altered neurogenesis in the hippocampus is a hallmark of early AD^6,7^.

Localization-based approaches have been intensively adopted in AD studies, such as *in vivo* mapping of molecular changes and neurodegeneration. These approaches have built much of current knowledge base of AD pathophysiology. However, their strength is limited with regard to investigation of neuronal and synaptic dysfunction^8^. A network perspective, on the contrary, has the potential to reveal the underlying dynamic communication between interconnected brain regions, and as a consequence, provide new intermediate phenotypes of pathology. Studies of connectivity between brain regions have grown steadily since the earliest study was published about 15 years ago^9,10^. Friston first established functional connectivity of brain activity between brain regions as the neuronal changes within one brain region related to another^11^. Functional connectivity analysis of positron emission tomography (PET) data has been intensely adopted in AD pathology studies. Drzezga *et al*. showed that there are significant disruptions of whole-brain connectivity in amyloid-positive patients with MCI in typical cortical areas that highly connected with multiple other brain regions, such as precuneus, strongly overlapping with regional hypometabolism^12^. A more recent fMRI and PET-based study showed that impaired visual working memory correlated with brain activity within the posterior parietal association cortex, prefrontal cortex, and thalamus in AD patients^13^.

PET imaging of beta-amyloid (Aβ) plaques with tracers such as [^11^C] PiB^14^, [^18^F] flutemetamol^15^, [^18^F] florbetaben^16^, [^18^F] AV-45^17^, enables *in vivo* measurement of fibrillar Aβ deposition, which is well established as an early indicator of AD pathology. Accurate Aβ concentration measurement is critical for better understanding underlying disease mechanisms, developing prognostic techniques and identifying compatible surrogate indicators for treatment monitoring.

The spatial resolution of PET is relatively low, typically between 5 and 6 mm full-width at half-maximum (FWHM). The partial volume effect (PVE) is a phenomenon that degrades the quantitative accuracy of PET images. Because of PVE, the intensity of a particular voxel reflects the tracer concentration not only of the tissue within that voxel but also the surrounding area. The impact of PVE on Aβ PET images becomes more complicated. It is reported that the PET signal observed in white matter of both cortical and subcortical regions mainly comes from non-specific tracer binding^14^. Hence, the quantification based on raw PET images provides an inaccurate representation of Aβ deposition without appropriate partial volume correction (PVC).

PVC techniques are designed to correct the spillover effect caused by the poor spatial resolution of PET images^18,19^. The effect of resolution can be modeled as a convolution of the true image with a three-dimensional point-spread function (PSF). The PSF of a PET system varies spatially. In the case of brain imaging, since the distance from the cortex to the center of the imaging field is typically no more than 10 cm, the PSF is thought not to increase in size significantly^20^ and the variation should not affect the correction for PET images considerably^21^. Several algorithms have been proposed for correction of PVE in PET, such as Meltzer’s method^22^, the Müller-Gärtner (MG) method^23^ and the geometric transfer matrix (GTM) method^24^. In particular, Meltzer’s method uses two-component PVC which defines two categories of tissue, i.e. brain and non-brain tissue, and compensates the underestimated signal caused by PVE in non-brain tissue. Whereas, the MG PVC technique uses three components, i.e. gray matter (GM), white matter (WM) and cerebrospinal fluid to correct for PVEs in GM only. The GTM method is a volume-based PVC algorithm that uses anatomical data from an MRI scan. However, unlike the MG method, the GTM approach only corrects for spillover between all brain regions and recovers the true activity for multiple regions rather than providing a restored image. There is increasing interest in other approaches to PVC recently, such as iterative image-based deconvolution techniques^25–27^, wavelet-based PVC^28–30^ and reconstruction-based PVC^31,32^. It is worth mentioning that accounting for PSF during image reconstruction theoretically reduce PVEs in the reconstructed image, it does not completely remove PVEs^33^.

Previous research works have also investigated the impact of PVC in Aβ plaques PET image quality, for example, the estimated regional standard uptake value ratios (SUVRs) in a longitudinal study^34^. However, those studies focus on direct measurements of tracer density either cross-sectional or longitudinal. However, there is no consensus regarding whether PVC is necessary for quantitative PET analysis and, if so, what processing pipeline of correction should be used. We believe such uncertainty is attributable to the limited understanding of the impact of PVC on Aβ PET images. In this study, we developed a method to evaluate the impact of PVC on quantitative Aβ PET imaging in a network perspective. Additionally, we also investigate the network properties of different stages during the pathological progress of AD. Furthermore, we used three different classifiers to perform classification tests between uncorrected and corrected images under same parameter settings to confirm that after partial volume correction, images from different disease stages are better distinguishable.

## Methods

### Participants

This study used data from subjects consisted of 97 normal cognition (NC), 96 early mild cognitive impairment (EMCI), 129 late mild cognitive impairment (LMCI) and 91 Alzheimer’s disease (AD) in the Alzheimer’s Disease Neuroimaging Initiative (ADNI). All ADNI sites must be appropriately licensed through appropriate state or federal agencies to receive and use AV-45 prior to imaging. All participants provided informed consent and were studied under protocols approved by Institutional Review Board (IRB) and radiation safety committee (RSC). For more up-to-date information, please refer to www.adni-info.org.

All subjects in our study had at least one PET scan every year with a diagnosis of cognitive status that is consistent with AD, LMCI, EMCI, or stable NC. EMCI and LMCI are clinical concepts that characterize cognitive stages intermediately between normal aging and AD. Please refer to Table 1 for a summary of the demographic and clinical data of the subjects. (The full subject list, by code names and their detailed information can be found in supplemental materials and can be used to retrieve the PET and other clinical data from the ADNI website hosted by LONI, http://adni.loni.usc.edu/.) AV-45 and FDG PET imaging is performed on all newly enrolled participants on two separate days (minimum 12-day time lapse). Scans are performed within two weeks before or two weeks after the in-clinic assessments at baseline and at the follow-up visit, 24 months after baseline.

**Table 1:** Group demographic and clinical summary for each cohort.

Group	Sample Size	Avg. Age (SD^a^)	Avg. Edu (SD)	Avg. CDR^b^ (SD)	Avg. MMSE^c^ (SD)
NC	97 (58 Female)	73.894(6.03)	16.530(2.46)	0.025(0.11)	28.970(1.32)
EMCI	96 (49 Female)	70.482(7.16)	16.323(2.69)	1.255(0.77)	28.260(1.58)
LMCI	129 (65 Female)	71.999(7.26)	16.076(2.73)	1.599(0.92)	27.466(1.78)
AD	91 (40 Female)	74.201(8.05)	15.739(2.68)	4.304(1.60)	23.163(2.15)

^a^SD indicates the standard deviation of the dataset.

^b^CDR indicates the clinical dementia rating, a five-point scale in which CDR-0 connotes no cognitive impairment, and then the remaining four points are for various stages of dementia: CDR-0.5 (very mild dementia), CDR-1 (mild dementia), CDR-2 (moderate dementia), and CDR-3 (severe dementia).

^c^MMSE indicates the mini-mental state examination, a 30-point questionnaire that is used extensively in clinical and research settings to measure cognitive impairment. Any score greater than or equal to 24 points (out of 30) indicates a normal cognition. Below this, scores can indicate severe (69 points), moderate (10–18 points) or mild (19–23 points) cognitive impairment.

### AV-45 PET acquisition

All ADNI subjects underwent PET scanning procedures between January 2005 and December 2007 to study cerebral glucose metabolism. Since subjects were recruited from different sites, PET images were acquired in different PET systems (for specific scanner types and correspondent subject number, please refer to Supplemental Table S1). Typically, subjects were injected with a dose of 370 MBq of AV-45 and rested comfortably in the room for approximately 30 minutes for the incorporation of AV-45 into the brain. According to the ADNI protocol, all PET scans were recorded with subjects’ eyes open. In general, scans started 50 minutes after injection. All sites performed 3D scanning consisting of four 5-minute frames. Images provided were corrected for Compton scatter, and measured attenuation correction based upon ‘transmission’ and ‘blank’ scans for those systems having rod sources, or by CT scan for those sites having a PET/CT scanner. Raw PET data were finally converted to DICOM file format for further processing^35^.

### T1-weighted MRI acquisition

Similar with AV-45 PET images, high resolution 3D T1-weighted MR images were acquired on different types of scanners (please refer to Supplemental Table S2 for specific scanner types and correspondent subject number). Imaging parameters of different types of scanners are selected and listed in Supplemental Table S3. All T1-weighted MRI data were acquired in the sagittal plane using an MP-RAGE pulse sequence. A baseline and follow-up scan were required for all healthy controls, with an average inter-scan interval of 3.3 months^36^. In our case, follow-up scans were excluded in network construction and statistical analysis.

### Preprocessing of PET and MR images

Since T1-weighted MRI and PET were acquired in their native acquisition space with different scanners, it is necessary to restore them in MNI152 standard space for further quantitative data analysis and network construction due to different modalities and scanners. MNI152 standard space refers to the space defined by a template generated at the Montreal Neurological Institute (MNI), where 152 stereotaxically normalized, T1-weighted scans were averaged to form a standard representation of the human brain. This standard template is distributed with the software library FSL^37–39^. Correspondence between MNI and acquisition space was determined using two-step affine registration. First, transformation parameters were determined to register the functional image (PET) in question to a structural image (T1-weighted MRI) obtained from the same subject. Then, registration parameters were obtained taking the structural image into MNI152 space. Both transformation matrices were concatenated to obtain a transform that takes the functional images into standard space. We used the FSL tool FLIRT^40,41^ to perform image registration.

### Partial volume correction on PET images

An image deblurring technique that uses the spatially invariant PSF of the scanner measured in the image space is utilized in our study^42^. In order to stabilize the deconvolution problem, this method uses the joint entropy (JE) between the PET image and a high-resolution MR image as an information-theoretic penalty. It is shown that JE method leads to faster convergence and a lower mean squared error as well as a smooth PET image with sharp boundaries consistent with MRI. Our aim was to develop an approach to evaluate the effectiveness of JE-based based partial volume correction (PVC) on AV-45 PET imaging and its impact on brain network analysis.

The method relies on a measured image-domain PSF which is tracer-specific. The cost function is the combination of a least squares data fidelity term and an anatomical prior term which penalizes the JE computed from the joint probability density function (PDF) of the PET and MR images, as showed below:

1 $${\rm{\min }}\,\sum _{x}\,{\Vert i(x)-(t\ast h)(x)\Vert }^{2}+\sum _{m}\,\sum _{n}\,{\delta }_{u}{\delta }_{v}p({u}_{m},{v}_{n})\mathrm{log}\,p({u}_{m},{v}_{n})$$

where $i$ is the true image, $t$ is the observed image, $h$ is the PSF function, $x$ is 3-D coordinates of an image voxel, $u$ and $v$ are the uniformly spaced discretized intensity ranges as vectors of the PET and MR images, respectively, ${\delta }_{u}$ and ${\delta }_{v}$ are the widths of the discrete intensity bins and $p({u}_{m},{v}_{n})$ is the ${ij}$ th element of the joint PDF computed on the regularly spaced intensity grid. The joint PDF of PET and MR images was approximated using a Parzen window technique. A gradient projection technique with a non-negativity constraint was adopted to minimize the combined cost function with a regularization parameter. The gradients for data fidelity and for JE penalty can be sufficiently computed by 2D FFT-based convolution. The step size was determined by means of a bent line search using the Armijo rule^43^.

### Definition of region of interest

**Figure 1:**
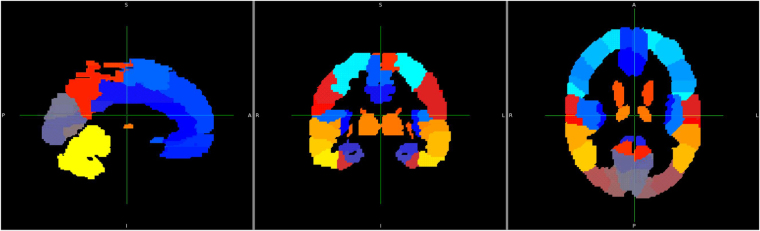
Regions of interest (ROIs). AAL template is aligned to MNI152 standard space.

We selected regions of interest (ROIs) using all 90 Automated Anatomical Labeling (AAL)^44^ map labels, as shown in Fig. 1. (For labels and details, please refer to Supplemental Table S4).

We generated probability masks for each ROI and computed average tracer intensity matrices of each group ${I}_{m\times n}=({i}_{{st}})$ as follows:

2 $${i}_{{st}}=\frac{{\sum }_{k=1}^{{k}_{s}}{w}_{k}{p}_{{tk}}}{{k}_{s}{\sum }_{k=1}^{{k}_{s}}{w}_{k}}$$

Here $m$ is the number of regions in functional network (in our case $m=90$), $n$ is the number of subjects, ${i}_{{st}}$ is the (*s*, *t*)th element of average tracer intensity matrix, which al*s*o indicates *st*h region of *t*th subject’s brain correlation network. ${k}_{s}$ is the total number of voxels in *s*th region of brain mask, ${w}_{k}$ is the tracer intensity of $k$ th voxel in brain mask (either 0 or 1) and ${p}_{{tk}}$ is the tracer intensity of $k$ th voxel of $t$ th subject.

### Network construction

A brain network is defined by a collection of nodes (vertices), and edges (links) between pairs of nodes. Nodes in large scale brain networks usually represent brain regions, while links represent anatomical, functional, or effective connections, depending on the dataset. In our study, brain networks are represented by their correlation matrices. The Pearson’s linear correlation coefficients ${\rm{C}}$ of each intensity matrix were computed using the average tracer intensity matrix. The percentage of positive elements in correlation matrices in NC, EMCI, LMCI and AD group is 100%, 99.98%, 99.03% and 98.53% respectively. We replaced the negative values in correlation matrices with zero. Rows and columns in correlation matrices denote nodes of 90 selected brain regions. Matrix entries denote edges that connect pairs of nodes, whose value indicate the correlation strength of the nodes.

To minimize the inter-subject difference, the standard deviation and the mean value of each column of network connectivity matrix was standardized (standardize *z*-score) so that each column of the connectivity matrix has mean of 0 and standard deviation of 1, as follows:

3 $${z}_{{ij}}=\frac{{x}_{{ij}}-E[{x}_{i}]}{\sigma ({x}_{i})}$$

where ${x}_{{ij}}$ is the $({\rm{i}},{\rm{j}})$ element in the connectivity matrix, $E[{x}_{i}]$ is the estimated expectation of each column and $\sigma ({x}_{i})$ is the estimated standard deviation. We then performed weight conversion and threshold on these matrices of normalized coefficients to finally construct our brain functional networks.

In this study, we first normalized the maximum connection value of all networks to 1 for inter-group analysis, i.e.

4 $${c}_{ij}=\frac{{z}_{ij}}{\mathop{\max }\limits_{i,j}\,{z}_{ij}}$$

After that we performed proportional threshold with proportion value ${\rm{p}}=0.1$ so that major global and local community structure in network can be preserved while most noise and false connections can be removed.

### Classification study

We used three different classifiers, matched subspace detection (MSD), linear discriminant analysis (LDA) and support vector machine (SVM), to perform the classification test on both the uncorrected and corrected images of EMCI and NC subjects. We used 10-fold cross-validation method to perform the tests: we first divide all subjects of EMCI and NC categories randomly into 10 groups, trained the classifiers with data from 9 groups and tested the classifiers using the last group. This training process was repeated 10 times with same parameter settings for both datasets so that each group was tested only once. Finally, we compared the average error rate and plotted the receiver operation characteristic (ROC) curve.

Matched subspace detection (MSD) is a classic method used to determine whether a multidimensional signal lies in a given linear subspace^45^. Hu *et al*. developed a MSD theory for signals derived from weighted graphs^46^. Graph Laplacian eigenvalues are regarded as frequencies of graph-signals and the signals are assumed to lie in a subspace spanned by the first few graph Laplacian eigenvectors that are associated with lower eigenvalues. Then the conventional matched subspace detection method is applied to this case.

## Results

### Image analysis

**Figure 2:**
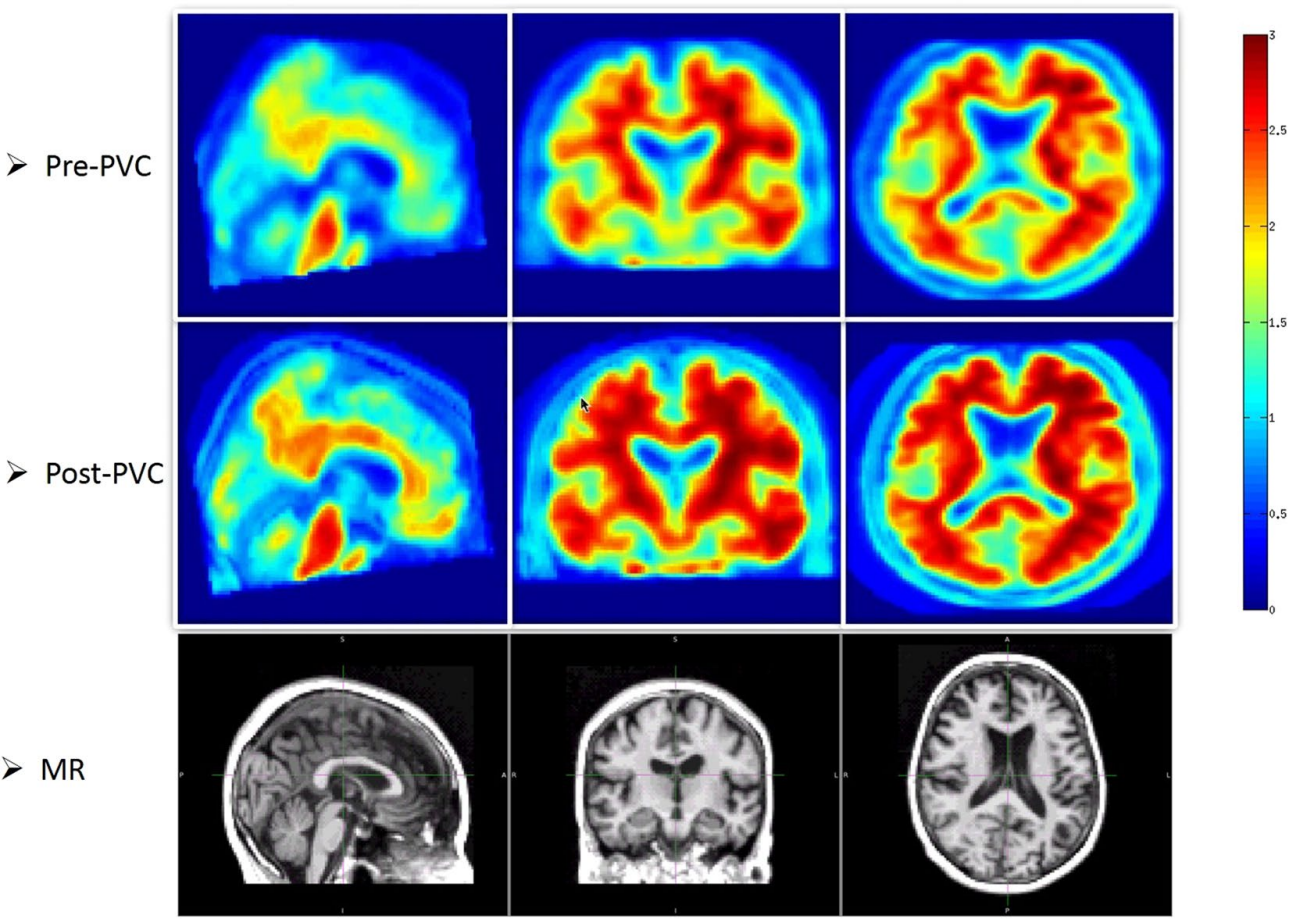
Representative PET images of pre- (top) and post-partial volume correction (middle). Anatomical prior (T1 weighted MR) information was used in PVC (bottom) of subject No. 003_S_4897. The subject is diagnosed with AD. The boundary of white matter and gray matter was strengthened.

After PVC was performed, the boundary of gray matter, white matter and cerebrospinal fluid (CSF) was strengthened, as shown in Fig. 2.

The effectiveness of our PVC method is examined by comparing improved quantification of other existing approaches to PVC in a clinical dataset. The cortex was parcellated into 90 regions as mentioned in Method section using the FreeSurfer software^47,48^ on all T1 MR images. Binary masks of each ROI were generated on a per-subject basis. The temporal frames of the PET scan from each subject were added together to create a sum image. All subsequent analysis of PET data was performed based on summed images. The PET image was rigidly registered to the anatomical image using the FLIRT tool in FSL^39^. For performance comparison of the PVC approach, we use the geometric transfer matrix (GTM) method, which is the most widely used PVC method in existing literature^24^.

First of all, the regional spread function (RSF) of each region was computed as the integration of the system’s PSF over each region, as showed below:

5 $$RS{F}_{i}(r)={\int }_{{D}_{i}}h(r,r^{\prime} )dr^{\prime} $$

here $i$ is the index of the region, $r$ and ${r}^{^{\prime} }$ are 3-D vectors in image and object reference space, respectively, ${D}_{i}$ is the space domain of $i$ th region and $h$ is the PSF of the system. Hence the mean value observed within each ROI can be written as weighted summation of mean value of all regions, as showed below:

6 $${t}_{j}=\frac{1}{{n}_{vox}}\sum _{i=1}^{N}{T}_{i}{\int }_{RO{I}_{i}}RS{F}_{i}(r)dr$$

here ${T}_{i}$ is the mean value of $i$ th region in true image and ${t}_{j}$ is the mean value of $j$ th region in observed image, ${n}_{{vox}}$ is the total number of voxels in ${{ROI}}_{j}$ and $N$ is the total number of regions in PET image. Equation (6) can be rearranged as below:

7 $${t}_{j}=\sum _{i=1}^{N}{\omega }_{{ij}}{T}_{i}$$

where weighting factors ${\omega }_{{ij}}$ can be expressed as below:

8 $${\omega }_{ij}=\frac{1}{{n}_{vox}}{\int }_{RO{I}_{j}}RS{F}_{i}(r)dr$$

The weighting factors of that equation represent the contribution of each region to any other region and we can rewrite equation (7) in a matrix form:

9 $$t={\rm{\Omega }}T$$

where $t$ is a vector of observed mean activities within each ROI, $T$ is a vector of true mean activities within each ROI and ${\rm{\Omega }}$ is the matrix of regional transfer coefficients. Mean value of true activity within ROIs can be recovered by computing each GTM coefficients ${\omega }_{{ij}}$ and solving the system of linear equations.

GTM correction was performed on all subjects and four information matrices were constructed based on different groups. Network matrices were then generated based on Pearson’s correlation. Negative elements were replaced with zeros and a proportional threshold of $p=0.1$ was then applied to the network matrices. Similar procedure was also applied to our PVC method.

**Figure 3:**
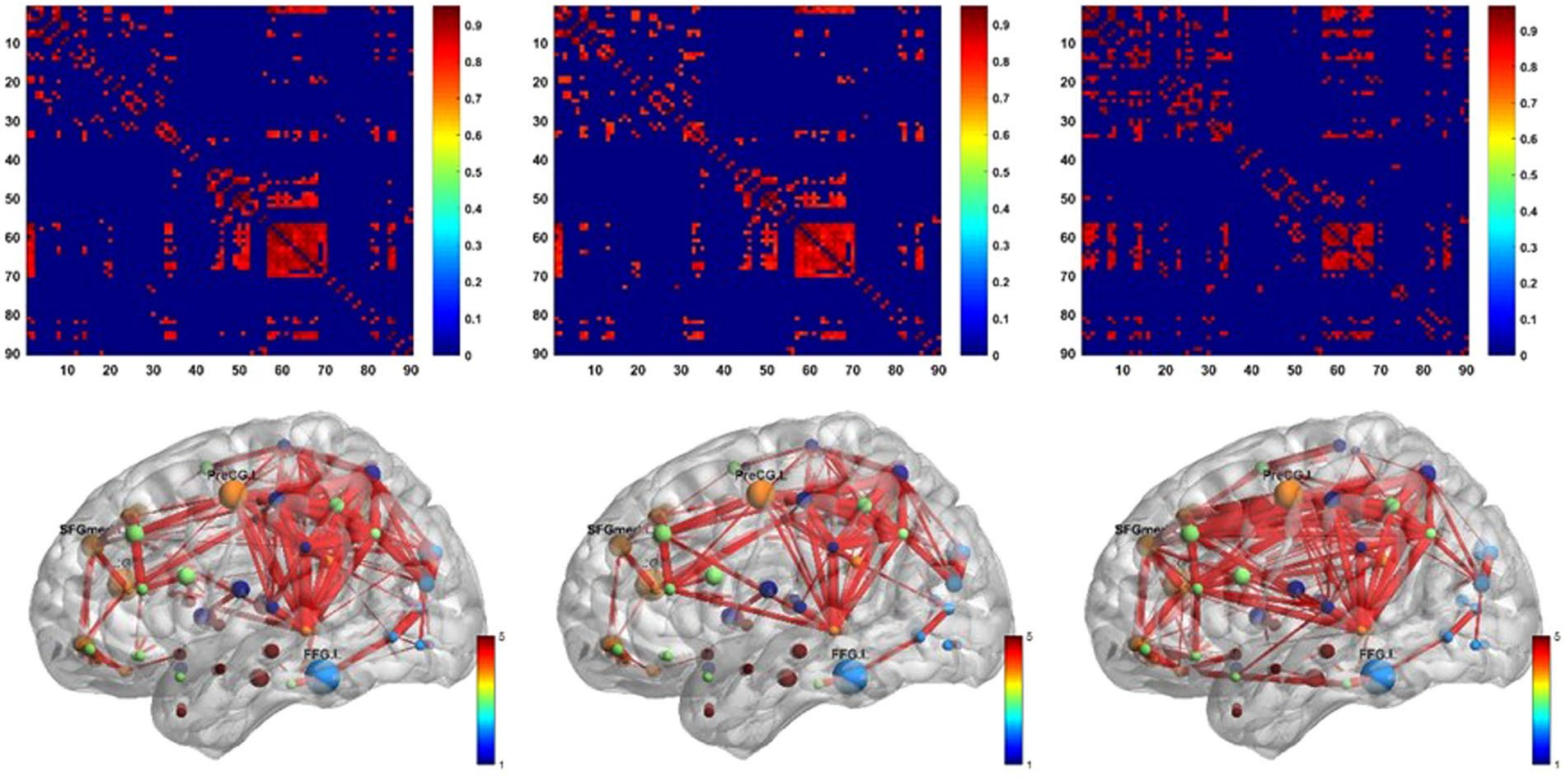
Network matrix (top row) and constructed brain network (bottom row) of NC group. From left to right: networks based on raw data, data corrected using GTM method and data corrected using JE-based method.

Figure 3 shows NC group network matrix constructed using raw data, GTM-based corrected data and JE-based corrected data. Our method significantly increased heterogeneity in network matrix compared to GTM method, especially in the prefrontal cortex, where existing edges in the original network were strengthened and some invisible connections were revealed.

### SUVR analysis

In this section, we present our findings from the analysis of SUVR change after partial volume correction. The quantitative accuracy of voxel-level recovery of our PVC method was evaluated by comparing our method with classic a deconvolution PVC method, modified Van Cittert (VC) iteration method^49^. The corrected image $t$ is estimated by minimizing the least squares (LS) criterion:

10 $$\sum _{x}{\Vert i(x)-(t\ast h)(x)\Vert }^{2}$$

where $i$ is the observed image, $h$ is the PSF and $x$ is the 3-D coordinate of an image voxel. The steepest descent scheme was applied to formulate an iterative rule to solve such deconvolution problem:

11 $$\begin{array}{rcl}{t}_{0}(x) & = & i(x);\\ {t}_{k+1}(x) & = & {t}_{k}(x)+\alpha (h\ast (i-h\ast {t}_{k}))(x)\end{array}$$

where $\alpha$ is the step length of iterations and we set $\alpha =1.5$ in this work. In addition, the following termination condition was applied to enhance the convergence of the algorithm:

12 $$\frac{\sqrt{{\sum }_{x}{({t}_{k+1}(x)-{t}_{k}(x))}^{2}}}{\sqrt{{\sum }_{x}i{(x)}^{2}}}\, < \,0.01$$

The SUVR images, in this context, are PET images (both corrected and uncorrected) normalized by the mean cerebellar GM uptake value, found using the cerebellar GM mask generated in the FreeSurfer software. Regional mean values were calculated for corrected and uncorrected SUVR images for ROIs defined as follows: putamen, frontal lobe and parietal lobe. The caudate was chosen as an example of striatal regions which in previous studies have shown increased A ${\rm{\beta }}$ protein uptake in AD. The putamen was chosen as an example of a subcortical region that can have elevated A ${\rm{\beta }}$ protein uptake in AD. The frontal and parietal lobe were also chosen as larger gray-matter volumes. To improve robustness, a 10% interquartile mean value was calculated for each region as follows:

13 $${x}_{{IQM}}=\frac{9/10}{n}\sum _{i=\lfloor 0.05n+1\rfloor }^{\lceil 0.95n\rceil }{x}_{i}$$

The standard deviation and coefficient of variance were calculated across subjects within a group (CoV_s_).

PVC increased cortical SUVR measurements, with the largest increases seen in AD subjects (please refer to Table 2). This is in line with previous findings that the quantification of cortical SUVR is severely affected by PVEs^19^. However, our method showed less increased bias in frontal and parietal regions compared to modified VC method. Variability in MCI group was more significant than that in other subject groups for cortical regions. The MCI group tended to exhibit a bipolar distribution in cortical regions, which indicates that MCI subjects appear to be similar with either AD or NC subjects. This is in line with previous findings that different types of Aβ PET tracer are diagnostically inconsistent among MCI subjects^50^. The EMCI group tends to have highest caudate uptake among four groups, which may be due to image noise and non-specific binding in white matter. Our method showed lower mean value in EMCI group compared to modified VC method. Moreover, variability in caudate of NC, LMCI and AD group was reduced using our method.

**Table 2:** SUVR (mean ± SD (CoV_s_)) of brain regions.

Brain Region	Uncorrected/LSPVC/JEPVC	Groups			
		NC	EMCI	LMCI	AD
Frontal lobe	Uncorrected	0.41 ± 0.12(0.29)	0.39 ± 0.12(0.31)	0.36 ± 0.10(0.28)	0.36 ± 0.11(0.18)
	JEPVC	0.43 ± 0.12(0.28)	0.40 ± 0.11(0.28)	0.37 ± 0.10(0.30)	0.37 ± 0.10(0.21)
	LSPVC	0.45 ± 0.13(0.29)	0.46 ± 0.12(0.26)	0.39 ± 0.12(0.31)	0.39 ± 0.12(0.24)
Parietal lobe	Uncorrected	0.42 ± 0.12(0.29)	0.38 ± 0.12(0.32)	0.35 ± 0.11(0.31)	0.36 ± 0.12(0.14)
	JEPVC	0.44 ± 0.12(0.27)	0.39 ± 0.11(0.33)	0.36 ± 0.12(0.33)	0.36 ± 0.11(0.16)
	LSPVC	0.45 ± 0.13(0.31)	0.43 ± 0.13(0.30)	0.45 ± 0.12(0.25)	0.39 ± 0.12(0.24)
Caudate	Uncorrected	0.38 ± 0.11(0.28)	0.42 ± 0.10(0.24)	0.38 ± 0.11(0.29)	0.41 ± 0.11(0.27)
	JEPVC	0.35 ± 0.09(0.26)	0.37 ± 0.10(0.28)	0.35 ± 0.10(0.30)	0.38 ± 0.10(0.28)
	LSPVC	0.35 ± 0.10(0.29)	0.39 ± 0.10(0.26)	0.35 ± 0.11(0.31)	0.37 ± 0.11(0.29)
Putamen	Uncorrected	0.55 ± 0.11(0.19)	0.52 ± 0.11(0.21)	0.50 ± 0.10(0.21)	0.50 ± 0.10(0.20)
	JEPVC	0.51 ± 0.11(0.21)	0.50 ± 0.10(0.20)	0.45 ± 0.10(0.22)	0.46 ± 0.10(0.22)
	LSPVC	0.52 ± 0.11(0.21)	0.53 ± 0.11(0.21)	0.56 ± 0.10(0.18)	0.58 ± 0.11(0.19)

### Network measurements

In this section, we constructed population-based brain networks of different stages in AD pathological progression (NC, EMCI, LMCI, and AD) using both corrected and uncorrected images. We have experimented two different ways to construct the network: Pearson’s linear correlation and mutual information (MI). Pearson’s linear correlation coefficient indicates the linear relationship between different brain regions while MI is a measure of the mutual dependence between the two variables. Since the network structure looks extremely similar under two conditions, the linear correlation method was used in all subsequent analysis. After network matrix is computed, we applied a proportional threshold $p=0.1$ to the networks. Finally, the network properties of both corrected and uncorrected images of different groups were calculated.

**Figure 4:**
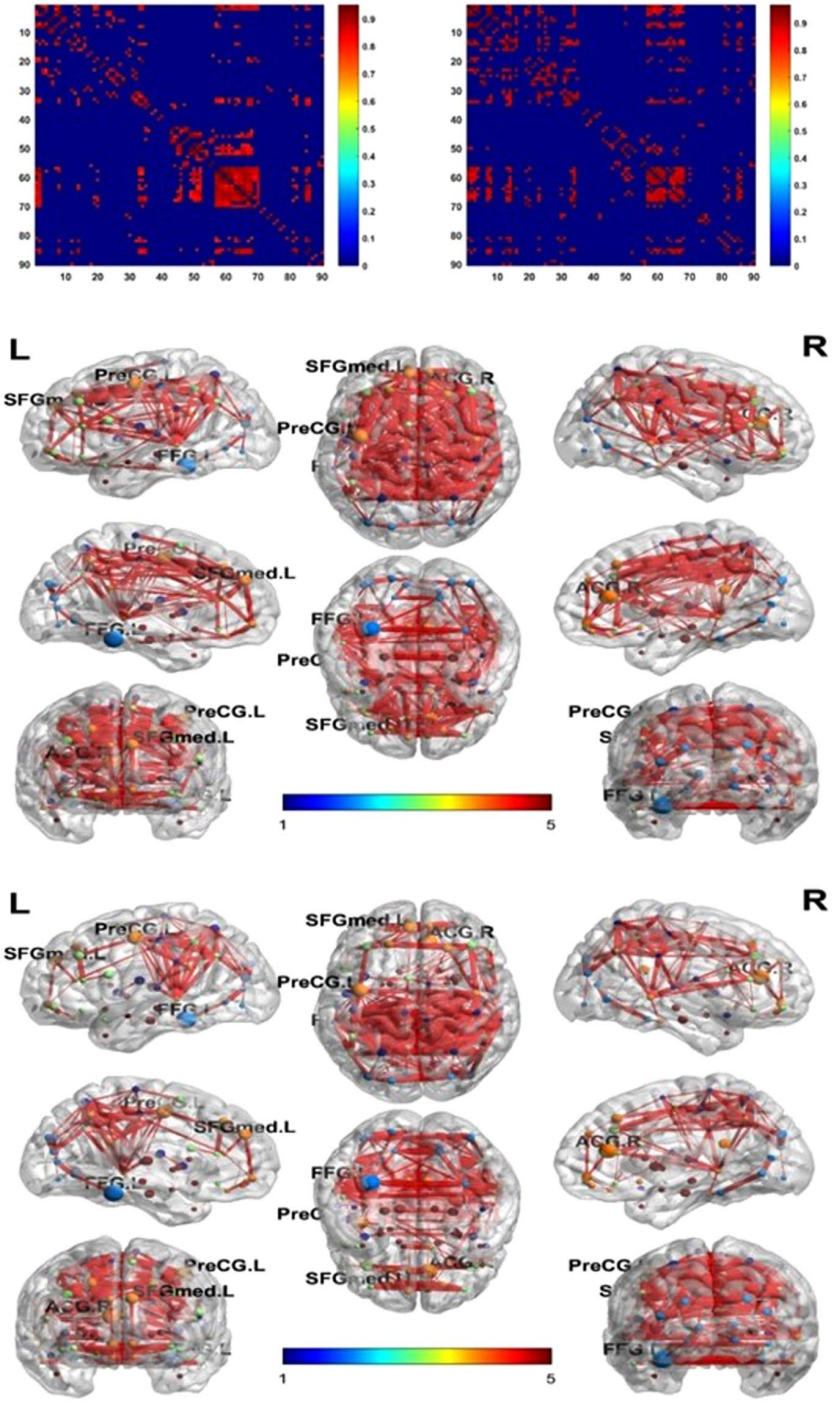
Network matrix of NC group for both PV-uncorrected (left) and corrected (right) images. Network structure was visualized using BrainNet Viewer for PV-uncorrected(top) and corrected (bottom) network.

We first visualized the network matrices to observe the differences of network structure after PVC. The BrainNet Viewer was used for the visualization (http://www.nitrc.org/projects/bnv/)^51^. After PVC, some inter-regional edges were recovered on all groups (please refer to Fig. 4 for an example of NC group), which indicated that PVC may reveal information of brain connectivity.

**Figure 5:**
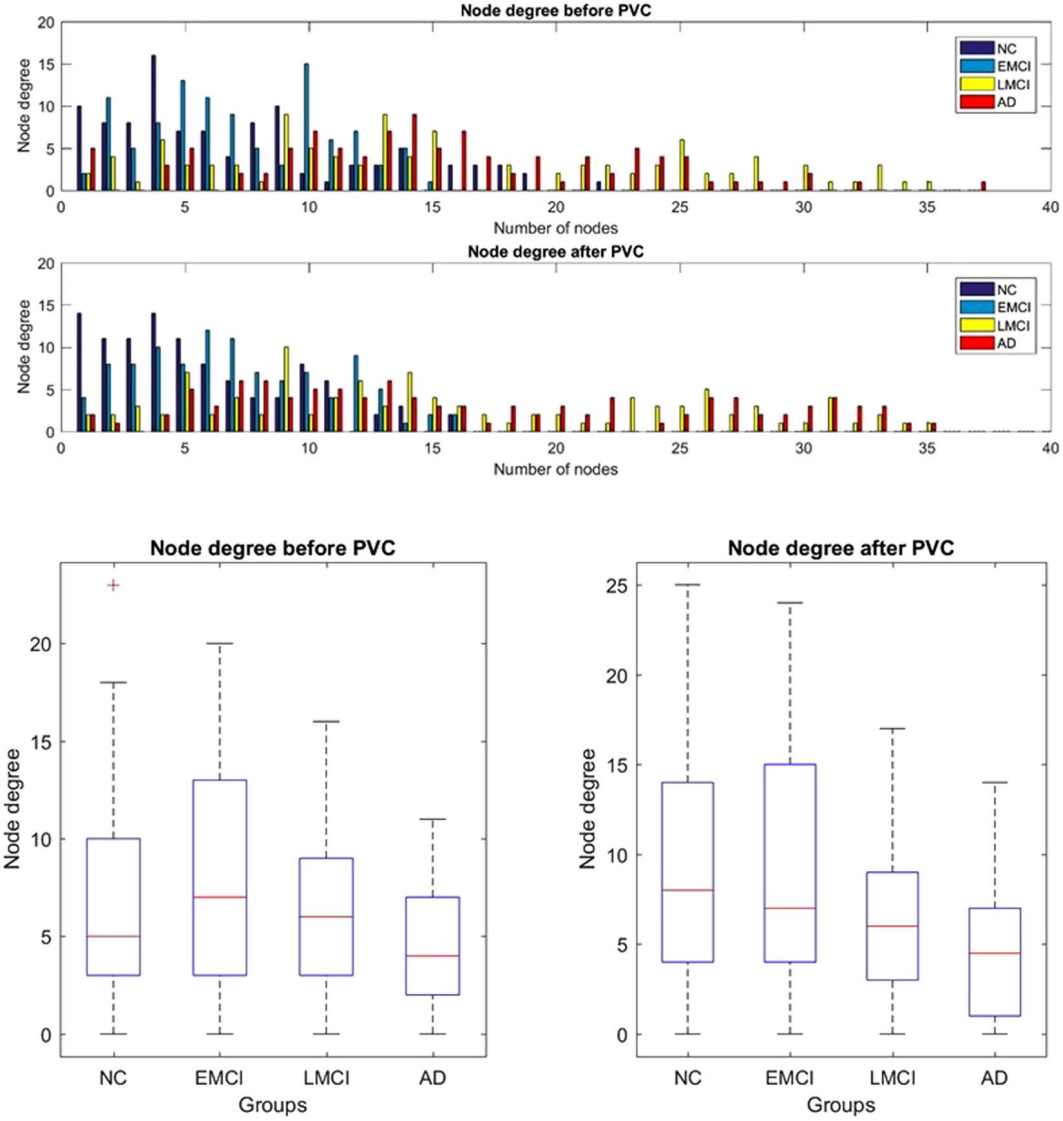
Histogram plots (top) and boxplots (bottom) of network degree distribution of four groups for both corrected and uncorrected images. Degree distribution of NC group increased significantly after PVC.

Network properties (node degree, network density, clustering coefficient, global effiency, maximum modularity, and node betweenness centrality) were then computed (please refer to Tables 3, 4 and Fig. 5). Node degree. The node degree is the number of links connected to the node. Connection weights are ignored in our calculations. In this study, we binarized the connectivity matrices and computed the sum of each row to form the node degree vector of each group. Network density. The network density is the fraction of present connections to possible connections, which can be represented as follows:

14 $$d=\frac{2K}{{N}^{2}\,-\,N}$$

here $N$ indicates the number of vertices (in our study $N=104$), $K$ indicates the number of edges and $d$ from 0 to 1 is the density of the network; higher $d$ indicates more intense network. Connection weights are ignored in calculations. In this study, we computed the number of vertices and edges and further the density of networks. Global efficiency. The global efficiency is the average of inverse shortest path length, and is inversely related to the characteristic path length. Clustering coefficient. The clustering coefficient is the fraction of triangles around a node and is equivalent to the fraction of node’s neighbors that are neighbors of each other. Maximum modularity. The optimal community structure is a subdivision of the network into non-overlapping groups of nodes in a way that maximizes the number of within-group edges, and minimizes the number of between-group edges. The modularity measure $Q(p)$ for a given partition $p$ of the functional human brain network is defined as follows:

15 $$Q(p)=\sum _{i=1}^{N}[\frac{{p}_{i}}{P}-{(\frac{{d}_{i}}{2P})}^{2}]$$

where $N$ is the number of modules, $P$ is the number of connections in the network, ${p}_{i}$ is the connections between nodes in module $i$ and ${d}_{i}$ is the sum of the degrees of nodes in module $i$ ^52^. The modularity is a statistic that quantifies the degree to which the network may be subdivided into such clearly delineated groups. The value of modularity varies from 0 to 1; higher value indicates more robust the subdivision. In practice, a modularity value above 0.3 is a good indicator of significant modules in a network^53^. Node betweenness centrality. Node betweenness centrality is the fraction of all shortest paths in the network that contain a given node. Nodes with high values of betweenness centrality participate in a large number of shortest paths and may be a hub node of the network.

**Table 3:** Network properties of different groups.

Metric	NC		EMCI		LMCI		AD	
	pre	post	pre	post	pre	post	pre	post
$D$	0.0633	0.0878	0.0794	0.0904	0.0620	0.0596	0.0414	0.0444
${E}_{{global}}$	0.2478	0.3109	0.2879	0.3229	0.2476	0.1827	0.1076	0.1378
${C}_{{wu}}$	0.3959	0.4865	0.5059	0.5195	0.4346	0.4222	0.3941	0.4248
${M}_{{\max }}$	0.5327	0.5301	0.5969	0.5582	0.6593	0.6692	0.7261	0.7290

$D$ indicates the network density, ${E}_{{global}}$ indicates global efficiency, ${C}_{{wu}}$ indicates the clustering coefficient, and ${M}_{{\max }}$ indicates the maximum modularity of network.

**Table 4:** Nodes with highest betweenness centrality.

Uncorrected	Corrected
Frontal_Inf_Tri_L^*^	Frontal_Inf_Tri_L^*^
Frontal_Med_Orb_R^*^	Frontal_Med_Orb_R^*^
Putamen_R^*^	Putamen_R^*^
Putamen_L^*^	Putamen_L^*^
Amygdala_R^*^	Amygdala_R^*^
Occipital_Mid_L^*^	Angular_R^*^
Angular_R^*^	Occipital_Mid_L^*^
Occipital_Mid_R	SupraMarginal_R
Cingulum_Ant_L	Calcarine_L
Temporal_Sup_R	ParaHippocampal_L
Temporal_Mid_R	Frontal_Inf_Oper_R
Insula_L	Frontal_Inf_Tri_R
Insula_R	Frontal_Med_Orb_L

*Indicates nodes with relative high betweenness centrality both in PV-uncorrected and corrected networks (sorted from highest to lowest).

Node degree distribution of NC group significantly increased after PVC (please refer to Fig. 5). There is a decreasing trend of node degree along the AD pathological progression.

It is reported that reduced network connectivity is a significant predictor of conversion to AD independently of global atrophy, and functional connectivity changes are related to memory deficits^54^. In our study, we found that progression of AD is accompanied with global density of functional brain network decreasing. The network density of NC and EMCI both increased significantly after PVC, which indicates some connections were weakened due to the blurriness of original PET images. NC group has a network density 25.4% lower than EMCI before PVC, and this ratio is reduced to 2.96% after PVC, which infers that PVC significantly recovered brain network in NC group. However, it is worth mentioning that the network density of LMCI decreased after PVC, which may imply that PVC strengthened the network difference between EMCI and LMCI groups.

Similar phenomena after PVC can also be found in global efficiency, clustering coefficient of networks, that is, PVC minimizes the network differences with regard to global and clustering properties between NC and EMCI while strengthened the network differences between EMCI and LMCI. Overall, there is a decreasing trend of each network measure along AD propagation. Previous study has shown a loss of small-world structure towards a more randomized network topology^55–58^, which is demonstrated by a reduction in the clustering coefficient values. Our network measurement indicates a reduction in the clustering coefficient from EMCI to LMCI.

However, NC group shows a lower clustering coefficient than EMCI even after PVC. Insignificant atrophy and inter-subject variance may attribute to the clustering coefficient difference between NC and EMCI. It should also be pointed out that AD group has higher clustering coefficient than LMCI after PVC. This may be attributed to some local increased intrinsic connectivity during AD revolution, where the basis for these connectivity increase is not well studied^59–61^. One possible explanation could be that they represent compensatory plasticity reorganization mechanisms^59–61^.

The maximum modularity of networks indicates significant modular functional brain network structure in all groups. The increasing trend from NC to AD suggest more robust modular structure with AD propagation. However, after PVC, the maximum modularity of brain network decreased in NC and EMCI while increased in LMCI and AD, which indicates that a portion of global connections in brain network of LMCI and AD may be attributed to false connection caused by blurriness of PET image.

Nodes with highest betweenness centrality were listed in Table 4 for both PV-uncorrected and corrected images. Putamen was known as a subcortical region that has elevated PiB uptake in AD^62^ and this is in line with our result that putamen is one of nodes with highest betweenness centrality in both PV-corrected and uncorrected networks.

### Classification study

In this section, we performed a classification study on different groups using both corrected and uncorrected AV-45 PET data. Three different classifiers (SVM, LDA, and MSD) were used in this study. We used 10-fold cross-validation method to train the classifiers and test it under same parameter settings for both datasets. The difference of error rate and the receiver operator characteristic (ROC) curve were then computed and plotted to illustrate the effectiveness of PVC from an image-classification perspective.

#### Support vector machine

The histogram of oriented gradients (HOG)^63^ is a feature descriptor used in computer vision and image processing for the purpose of object detection. HOG decomposes an image into small squared cells, computes a histogram of oriented gradients in each cell, normalizes the result using a block-wise pattern, and return a descriptor for each cell. In our study, the dataset of each group was randomly divided into two equal subsets and the HOG features of one subset was extracted using the VLFeat library^64^ and then trained in a support vector machine (SVM). After that, the other subset was tested under same parameter setting. We then calculated the scores of the tests and computed the error rate of the tests (ratio of error test results and all test results). We repeated this procedure 200 times for each classification study.

**Figure 6:**
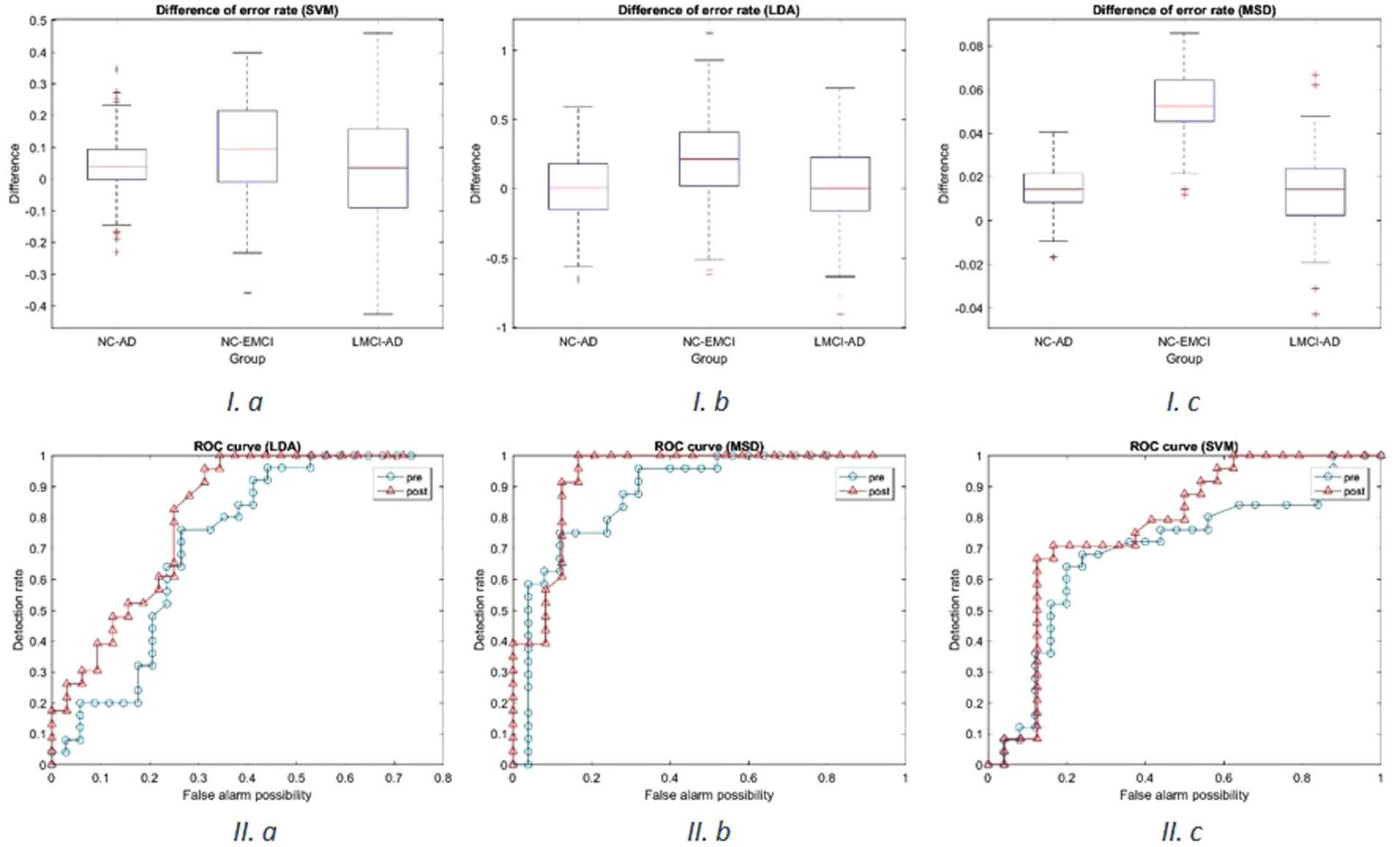
I. Difference of error rate. 10-fold cross-validation was repeated 200 times in classifier trained under same parameter setting with different training data (uncorrected and corrected images). The difference of error rate between uncorrected classifier and corrected classifier was computed and plotted. II. ROC curves for NC/EMCI classification. ROC curve is plotted as false alarm probability versus detection rate. The area below ROC curve is used to evaluate the performance of the classifier, larger area is correspondent to more robust performance.

After we performed the classification experiment of EMCI/NC case, AD/NC case and LMCI/AD case both on corrected and uncorrected images, we calculated the error rate difference of uncorrected and corrected images (since classifier trained with uncorrected images showed higher error rate in all cases), and plotted the distribution of such difference (please refer to Fig. 6I.a). A significant improvement was observed in NC/EMCI classification, which is more challenging due to similarity between two groups. There is limited improvement in other two classification cases; one possible reason may be the limitation of the method. ROC curve also suggests that classifier trained with corrected images has higher performance (please refer to Fig. 6II.a).

#### Linear discriminative analysis

Linear discriminant analysis (LDA) is a generalization of Fisher’s linear discriminant, a method used that characterizes or separates two or more classes of objects or events. In this study, since the scale of our data is limited, we used 10-fold cross-validation to evaluate the performance of the LDA classifier under same parameter settings given two training datasets respectively. We randomly divided our subjects into 10 groups, trained the LDA classifier with 9 groups and tested the LDA classifier on the remaining group. Similar with the SVM training process, such training and testing process was repeated 10 times so that all 10 groups of subjects is tested. The averaged ROC curve and the distribution of difference of error rate was computed and plotted after random 10-fold cross-validation was repeated for 200 times (please refer to Fig. 6I.b).

We observed from Fig. 6I.b that error rate significantly reduced after PVC in NC/EMCI study. Figure 6II.b illustrated that the LDA classifier trained with PVC images outperformed that trained with uncorrected images.

#### Matched subspace detection

We used 10-fold cross-validation to evaluate the performance of MSD classifier under same parameter setting given two training datasets respectively. We randomly divided our subjects into 10 groups, trained MSD classifier with 9 groups and tested MSD classifier on the remaining group. Such training and testing process was repeated 10 times so that all 10 groups of subjects is tested. The above 10-fold cross-validation process was repeated 200 times and the average error rate of 10 groups were recorded for both corrected and uncorrected training data. Then the difference of error rate was computed and plotted, as shown in Fig. 6I.c. It is worth mentioning that the difference of error rate was not improved as significantly as that in LDA and SVM, which may be attribute to higher accuracy of MSD classifier (mean error rate 12.78% in NC/AD classification, 17.67% in NC/EMCI classification and 14.16% in LMCI/AD classification), which results in limited improvement of performance.

We observed that the performance classifier on EMCI/NC classification was significantly improved when trained using PVC data (please refer to Fig. 6II.c).

## Discussion

PVC was evaluated using a clinical AV-45 dataset containing subjects from different stages of AD progression. In the SUVR analysis, the inter-voxel variability within each region after PVC is affected by two factors. It may increase due to amplification of noise or decrease due to reduced sub-regional bias. The final result depends on the balance of two effects. The increased regional variability in our PVC method indicates that PV correction may amplify noise in PET quantification. We speculate that the noise amplification may be attributable to PET to MR registration and segmentation of anatomical MR data^65^. Moreover, we found that the variability is bound to reduce if we increase the regularization parameter.

The average node degree for NC is less than EMCI when the brain network is constructed using PV-uncorrected images. This can be explained by examining the inter-subject variance of SUVR in NC group since the signal observed in white matter mainly comes from non-specific binding and it is impossible for the spatial resolution of PET permitted imaging gray matter without partial volume contributions from white matter. However, the node degree distribution of the NC group significantly changed after PVC and the average degree of NC is more than the EMCI group, which may indicate that the partial volume contributions of white matter has been removed in the network. Moreover, it should be pointed out that the node degree showed a decreasing trend along AD progression. These results indicate that disruption of functional connectivity may represent early functional consequences of emerging molecular Alzheimer’s disease pathology, evolving prior to clinical onset of dementia.

Based on the betweenness centrality of nodes, we can infer that some regions may function as potential hubs within a brain network. By comparing networks constructed both from PV-uncorrected and corrected images we found some common nodes with high betweenness centrality. Putamen has been reported as a subcortial region with an PiB elevation uptake in AD, which also indicated high interaction in our network analysis. The other regions, such as frontal lobe and temporal lobe, may also play an important role in Aβ deposition process of AD. It is worth mentioning that after visualization of brain networks, some edges connected cortial local communities were found recovered after PVC, which may indicate that PVEs severely affected the cortical SUVR quantification.

EMCI/NC classification is challenging. It has been reported that normal elderly controls may also have high Aβ binding in the PiB-PET image^17^, which makes the image of NC and EMCI much more homogeneous. However, we found that the average error rate of classification reduced significantly after PVC using different classifiers. This can be explained by enhanced image features within each group after the removal of partial volume contribution. It should be pointed out that such reduction in error rate of AD/NC and LMCI/AD classification is not as obvious as NC/EMCI classification. This suggests that the image feature of LMCI and AD may be distinguishable enough even without PVC so that there is limited space for improvement of PVC in these groups.

## Conclusion

A network-based approach for evaluating the impact of PVC on AV-45 PET imaging was developed and applied to NC, EMCI, LMCI and AD subjects. PVC compensates for partial volume effects, which, if uncorrected, lead to spill-in and spill-out of estimated activity between a voxel or region of interest (ROI) and its neighbors. A theoretical cost of PVC is that it increases the noise of PET image due to uncertainties in image registration and segmentation. However, our result demonstrates that PVC improved the network structure of each group and revealed the pathological progression along AD revolution. Furthermore, PVC also improves the image features of each group, as illustrated in the classification study. Therefore, we recommend PVC be performed in all AV-45 PET studies, although standardization of the PVC technique may be needed to compare studies across different groups.

## Electronic supplementary material

Supplementary materials
