# Supplementary material for: Partial volume correction for PET quantification and its impact on brain network in Alzheimer’s disease
Source: Sci Rep. 2017 Oct 12;7:13035. doi: 10.1038/s41598-017-13339-7 (PMC5638902; doi:10.1038/s41598-017-13339-7)
Supplement: Supplementary file 1 — Supplementary materials [file 41598_2017_13339_MOESM1_ESM.pdf]

Partial volume correction for PET quantification and its impact on brain network in Alzheimer's disease

Jiarui Yang[1,2], Chenhui Hu[2], Ning Guo[2], Joyita Dutta[2,4], Lucia M. Vaina[1,2], Keith A. Johnson[2,3], Jorge Sepulcre[2,3], Georges El Fakhri[2,3], and Quanzheng Li[2,3,*]

[1]Boston University, Department of Biomedical Engineering, Boston, 02215, USA
[2]Massachusetts General Hospital, Department of Radiology, Boston, 02114, USA
[3]Harvard Medical School, Department of Radiology, Boston, 02115, USA
[4]University of Massachusetts Lowell, Department of Electrical and Computer Engineering, Lowell, 01854, USA
[*]corresponding.li.quanzheng@mgh.harvard.edu

Table S1. Number of subjects of all types of PET scanners used.

Manufacturer	Model Type	NC	EMCI	LMCI	AD
GE	Discovery RX	2	3	2	2
	Discovery LS	6	10	6	3
	Discovery STE	25	25	22	16
	Advance	4	6	2	3
SIEMENS/CTI	1094	5	2	10	4
	1093	5	0	11	1
	HR+	22	15	24	30
	LSO PET/CT HI-REZ	14	13	17	10
	HRRT	5	4	4	5
	SOMATOM	0	3	0	10
	ACCEL	0	0	1	0
PHILIPS	GEMINI TF TOF 16	8	9	13	0
	Allegro Body	4	2	4	2
	Guardian Body	1	0	0	0
	Ingenuity TF PET/CT	1	1	7	5

Table S2. Number of subjects of all types of MRI scanners used.

Manufacturer	Model Type	NC	EMCI	LMCI	AD
GE	SIGNA EXCITE	44	44	53	43
	SIGNA HDxt	3	4	5	4
	GENESIS_SIGNA	6	10	11	9
SIEMENS/CTI	SYMPHONY	17	3	28	7
	SONATA	13	16	7	5
	SONATA Vision	0	0	2	1
	AVANTO	2	3	9	4
	Trio Tim	1	2	0	7
	VERIO	1	2	0	1
PHILIPS	INTERA	14	7	9	10
	GYROSCAN INTERA	2	0	0	0
	INTERA ACHIEVA	1	5	4	0
	ACHIEVA	0	0	2	0

Table S3. Imaging parameters of all types of MRI scanners used.

Manufacturer	Model Type	Coil	FS(T)	FA (°)	Matrix Size	PS(mm)	T(mm)	TE(ms)	TR(ms)
GE	SIGNA EXCITE	8HR	1.5	8.0	256*256*166	0.937	1.2	3.924	8.916
	SIGNA HDxt	8HR	3.0	11.0	256*256*196	1.016	1.2	2.840	6.972
	GENESIS_SIGNA	HD	1.5	8.0	256*256*180	1.016	1.2	4.084	10.20
SIEMENS/CTI	SYMPHONY	PA	1.5	8.0	192*192*160	1.250	1.2	3.610	3000
	SONATA	HE	1.5	8.0	192*192*160	1.250	1.2	3.540	3000
	SONATA Vision	PA	1.5	8.0	192*192*160	1.250	1.2	3.540	2400
	AVANTO	PA	1.5	8.0	192*192*160	1.250	1.2	3.500	2400
	Trio Tim	PA	3.0	9.0	240*256*176	1.000	1.2	2.980	2300
	VERIO	PA	3.0	9.0	240*256*176	1.055	1.2	2.950	2300
PHILIPS	INTERA	SH	1.5	8.0	256*256*170	0.9375	1.2	4.005	8.615
	GYROSCAN INTERA	SH	1.5	8.0	256*256*170	0.9375	1.2	3.987	8.576
	INTERA ACHIEVA	8SH	1.5	8.0	256*256*170	0.9735	1.2	3.981	8.554
	ACHIEVA	8SH	3.0	9.0	256*256*170	1.000	1.2	3.137	6.767

FS indicates the field strength, FA indicates the flip angel, PS indicates the pixel space (all models' pixel space is square and thus is represented by its length of the side), T indicates the thickness, TE indicates the echo time, and TR indicates the repetition time.

Table S4. Regions of a brain functional network.

Index	Abbreviation	Full name
1	Precentral_L	Left precentral gyrus
2	Precentral_R	Right precentral gyrus
3	Frontal_Sup_L	Left superior frontal gyrus
4	Frontal_Sup_R	Right superior frontal gyrus
5	Frontal_Sup_Orb_L	Left superior frontal gyrus, orbital part
6	Frontal_Sup_Orb_R	Right superior frontal gyrus, orbital part
7	Frontal_Mid_L	Left middle frontal gyrus
8	Frontal_Mid_R	Right middle frontal gyrus
9	Frontal_Mid_Orb_L	Left middle frontal gyrus, orbital part
10	Frontal_Mid_Orb_R	Right middle frontal gyrus, orbital part
11	Frontal_Inf_Oper_L	Left inferior frontal gyrus, pars opercularis
12	Frontal_Inf_Oper_R	Right inferior frontal gyrus, pars opercularis
13	Frontal_Inf_Tri_L	Left inferior frontal gyrus, pars triangularis
14	Frontal_Inf_Tri_R	Right inferior frontal gyrus, pars triangularis
15	Frontal_Inf_Orb_L	Left inferior frontal gyrus, pars orbitalis
16	Frontal_Inf_Orb_R	Right inferior frontal gyrus, pars orbitalis
17	Rolandic_Oper_L	Left Rolandic operculum
18	Rolandic_Oper_R	Right Rolandic operculum
19	Supp_Motor_Area_L	Left supplementary motor area
20	Supp_Motor_Area_R	Right supplementary motor area
21	Olfactory_L	Left olfactory cortex
22	Olfactory_R	Right olfactory cortex
23	Frontal_Sup_Medial_L	Left medial frontal gyrus
24	Frontal_Sup_Medial_R	Right medial frontal gyrus
25	Frontal_Med_Orb_L	Left medial orbitofrontal cortex
26	Frontal_Med_Orb_R	Right medial orbitofrontal cortex
27	Rectus_L	Left gyrus rectus
28	Rectus_R	Right gyrus rectus
29	Insula_L	Left insula
30	Insula_R	Right insula
31	Cingulum_Ant_L	Left anterior cingulate gyrus
32	Cingulum_Ant_R	Right anterior cingulate gyrus
33	Cingulum_Mid_L	Left midcingulate area
34	Cingulum_Mid_R	Right midcingulate area
35	Cingulum_Post_L	Left posterior cingulate gyrus
36	Cingulum_Post_R	Right posterior cingulate gyrus
37	Hippocampus_L	Left hippocampus
38	Hippocampus_R	Right hippocampus
39	ParaHippocampal_L	Left parahippocampal gyrus
40	ParaHippocampal_R	Right parahippocampal gyrus
41	Amygdala_L	Left amygdala
42	Amygdala_R	Right amygdala
43	Calcarine_L	Left calcarine sulcus
44	Calcarine_R	Right calcarine sulcus
45	Cuneus_L	Left cuneus
46	Cuneus_R	Right cuneus
47	Lingual_L	Left lingual gyrus
48	Lingual_R	Right lingual gyrus
49	Occipital_Sup_L	Left superior occipital
50	Occipital_Sup_R	Right superior occipital
51	Occipital_Mid_L	Left middle occipital gyrus

Continued on next page

52	Occipital_Mid_R	Right middle occipital gyrus
53	Occipital_Inf_L	Left inferior occipital cortex
54	Occipital_Inf_R	Right inferior occipital cortex
55	Fusiform_L	Left fusiform gyrus
56	Fusiform_R	Right fusiform gyrus
57	Postcentral_L	Left postcentral gyrus
58	Postcentral_R	Right postcentral gyrus
59	Parietal_Sup_L	Left superior parietal lobule
60	Parietal_Sup_R	Right superior parietal lobule
61	Parietal_Inf_L	Left inferior parietal lobule
62	Parietal_Inf_R	Right inferior parietal lobule
63	SupraMarginal_L	Left supramarginal gyrus
64	SupraMarginal_R	Right supramarginal gyrus
65	Angular_L	Left angular gyrus
66	Angular_R	Right angular gyrus
67	Precuneus_L	Left precuneus
68	Precuneus_R	Right precuneus
69	Paracentral_Lobule_L	Left paracentral lobule
70	Paracentral_Lobule_R	Right paracentral lobule
71	Caudate_L	Left caudate nucleus
72	Caudate_R	Right caudate nucleus
73	Putamen_L	Left putamen
74	Putamen_R	Right putamen
75	Pallidum_L	Left globus pallidus
76	Pallidum_R	Right globus pallidus
77	Thalamus_L	Left thalamus
78	Thalamus_R	Right thalamus
79	Heschl_L	Left transverse temporal gyrus
80	Heschl_R	Right transverse temporal gyrus
81	Temporal_Sup_L	Left superior temporal gyrus
82	Temporal_Sup_R	Right superior temporal gyrus
83	Temporal_Pole_Sup_L	Left superior temporal pole
84	Temporal_Pole_Sup_R	Right superior temporal pole
85	Temporal_Mid_L	Left middle temporal gyrus
86	Temporal_Mid_R	Right middle temporal gyrus
87	Temporal_Pole_Mid_L	Left middle temporal pole
88	Temporal_Pole_Mid_R	Right middle temporal pole
89	Temporal_Inf_L	Left inferior temporal gyrus
90	Temporal_Inf_R	Right inferior temporal gyrus
